# Incident of Practice

**Published:** 1884-05

**Authors:** C. E. Francis


### INCIDENT OF PRACTICE.

BY C. E. FRANCIS, D. D. S., N. Y.

Mr. M., a New York merchant about sixty years of age, called some two months ago, complaining that for a long time he had experienced a decidedly unpleasant sensation all about the left side of his face, the gum and roof of his mouth feeling sore, and the soreness extending to the eye, the temple, and the ear. He had a wearied and care-worn look, was much depressed in spirit, and declared that he felt "miserable." He had for a long time been treated for "chronic catarrh," and was still under treatment, but with no improvement.

Removing a partial artificial denture, the roots of the left bicuspids were discovered in a badly decayed condition. These were extracted, and, through the alveolar openings of the second, considerable pus escaped. A probe introduced penetrated the antrum ; tepid water forced into the alveolus brought a quantity of vile-looking pus through the nostrils.

This case received daily treatment for two weeks. Tepid water, seasoned with chloride of sodium, was first injected, followed by carbolized water, to which was added a few drops of tinct. calendulæ, and the space kept open with tents of cotton coated with carbolized glycerine.* Indications of necrosis being discernible about the alveolar process, acid sulph. arom. was several times applied.

After the second week, treatment was less frequent, and now the case is all right, requiring no further care.

The patient is as well as ever, is bright and happy as possible, looks at least ten years younger, and declares he feels "twenty."

Query. Is dentistry only a mechanical art?

* Carbolized Vaseline is perhaps even better.

